# Identification and Regulation of TCRαβ^+^CD8αα^+^ Intraepithelial Lymphocytes in Murine Oral Mucosa

**DOI:** 10.3389/fimmu.2020.01702

**Published:** 2020-08-04

**Authors:** Ruiqing Wu, Dunfang Zhang, Peter Zanvit, Wenwen Jin, Hao Wang, WanJun Chen

**Affiliations:** ^1^Mucosal Immunology Section, National Institute of Dental and Craniofacial Research, National Institutes of Health, Bethesda, MD, United States; ^2^Beijing Tiantan Hospital, Capital Medical University, Beijing, China

**Keywords:** TCRαβ^+^CD8αα^+^, intraepithelial lymphocytes, unconventional immune cells, TGF-β, oral mucosa

## Abstract

TCRαβ^+^CD8αα^+^ intraepithelial lymphocytes (IELs) are abundant in gastrointestinal (GI) tract and play an important role in regulation of mucosal immunity and tolerance in the gut. However, it is unknown whether TCRαβ^+^CD8αα^+^ IELs exist in the oral mucosa and if yes, what controls their development. We here identified and characterized TCRαβ^+^CD8αα^+^ IELs from the murine oral mucosa. We showed that the number and function of TCRαβ^+^CD8αα^+^ IELs were regulated by TGF-β. We further revealed that oral TCRαβ^+^CD8αα^+^ IELs could be altered under systemic inflammatory conditions and by antibiotic treatment at the neonatal age of the mice. Our findings have revealed a previously unrecognized population of oral IELs that may regulate oral mucosal immune responses.

## Introduction

The gastrointestinal (GI) and respiratory tracts are both accessed through the oral cavity. The oral mucosa is a lining within the oral cavity which is composed of stratified squamous epithelium and the lamina propria (LP), and which surrounds various muscles, the teeth, the mandible, and the maxilla. Although it is a much thicker barrier than GI mucosa, which is composed of only a single layer of epithelium, oral mucosa is permeable, and fragile (1, 2). Numerous microorganisms and antigens and stimuli from exogenous substances (3) such as food undergo sophisticated regulation by immune responses in the oral mucosa. T cells especially intraepithelial T lymphocytes (IELs) are crucial for this defense (1). It has been reported that a subset of unconventional IELs expressing CD8αα (TCRαβ^+^CD8αα^+^) regulate intestinal inflammation (4). Many oral mucosal diseases have been shown to relate to impaired oral mucosal immune system and T-cell functions, such as *Candida albicans* infection (5), periodontitis (6), Sjögren's syndrome (7), and oral lichen planus (OLP) (8–12). Recently, it has also been demonstrated that the oral microbiota and immune status are associated to the pathogenesis of lichen planus and rheumatoid arthritis (8, 13, 14). However, the roles of oral IELs in healthy or pathological conditions remains unknown.

Here, we identified and characterized the phenotype and functions of TCRαβ^+^CD8αα^+^ oral IELs in mice. We found that TGF-β regulates the development/generation of oral CD8αα^+^ IELs. In addition, we observed that the number of oral CD8αα^+^ IELs could be changed by challenges of administration of systemic inflammatory agent imiquimod (IMQ) or by antibiotic treatment at the neonatal age of the mice. Our findings have revealed a previously unrecognized population of oral IELs that may regulate oral mucosal immune responses.

## Materials and Methods

### Animals

*Tgfb1*^−/−^, *Tgfbr1*^*f*/*f*^ *Cd4*-Cre^+^, *Abl/TGF*-β*1*-Transgenic mice and their littermate controls were generated as described previously (15–17). C57BL/6J mice were originally purchased from The Jackson Laboratory. All animals were bred and maintained under specific pathogen-free conditions in animal facilities of National Institutes of Health (NIH).

### Antibiotic Treatment

Depletion of microbiota was induced by antibiotic treatment. C57BL/6J mice received neonatal treatment with antibiotics using a combination of vancomycin (500 mg/mL, Mylan Institutional LLC) and polymyxin B (100 mg/mL, X-Gen Pharmaceuticals Inc.) during the first 3 weeks of their life (18). Antibiotic-treated neonates were weaned at 3 weeks and antibiotics were replaced with regular drinking water for a period of 8 weeks. All mice were euthanized at the age of 11 weeks (18). The antibiotics chosen are used in clinical practice to target Gram-positive (vancomycin) and Gram-negative (polymyxin B) bacteria, respectively. Antibiotics were given orally, and since both are poorly absorbed, systemic effect was avoided.

### Imiquimod (IMQ) Treatment

C57BL/6J mice were treated with 5% imiquimod (IMQ) cream (Fougera) on 2 cm^2^ shaved areas on the back and ears. Mice from the control group received pure petroleum jelly (Vaseline). The dosage of IMQ cream and pure petroleum jelly was 5 mg on ears and 60 mg on back skin. Treatments were applied daily for 6 consecutive days. Body weights were monitored daily during the treatment (18).

### Isolation of IELs From Intestines and Oral Mucosa

After euthanasia, the heart, liver, and tongue were perfused by PBS injection (Gibco Life Technologies). A precise dissection was performed to obtain tongue, buccal, oral floor, and gingival mucosa, taking care to avoid contamination by nasal associate lymphoid tissue (NALT), cervical draining lymph nodes (DLNs), salivary glands, muscles, and adipose tissue. Intestinal IELs were isolated following modified protocols as described previously (19, 20). The oral tissues were incubated at 37°C and mechanically stirred for 40 min in DMEM medium (Thermo Hyclone) supplemented with 4% (vol/vol) FBS (BenchMark), 5mM ethylenediaminetetraacetic acid (EDTA, Quality Biological Inc.) and 0.145 mg/ml of dithiothreitol (Fermented Life Sciences), after which tissues were washed thoroughly 3 times in DMEM medium with 2mM EDTA. The cell suspension was collected and filtered through 70 μm cell strainers (Corning Falcon). The cell pellet was centrifuged to precipitate.

### Isolation of LPLs From Oral Mucosa

After isolating IELs, tissues were minced into 2–4 mm^2^ pieces and digested using 5 mg/ml Collagenase IV/HBSS buffer (Gibco Life Technologies) at 37°C for 20 min. Tissues and suspension were mashed and filtered through 70 μm cell strainers (Corning Falcon). Cell pellet was centrifuged to precipitate. After centrifugation cells were proceeded to the gradient centrifugation using 30% (v/v), 55% (v/v), and 70% (v/v) Percoll/PBS (GE Healthcare/Gibco Life Technologies). Cell layers between 30% (v/v) and 55% (v/v), as well as 55% (v/v) and 70% (v/v), were collected and washed using DMEM. LPL cell pellet was centrifuged to precipitate.

### Antibodies and Flow Cytometry

Samples were stained with fluorescence-conjugated antibodies and analyzed using FACS Calibur and Cellquest software or using BD LSR Fortessa and FACS Diva software (Becton Dickinson). All data acquired from flow cytometry was analyzed using FlowJo software (TreeStar). The following antibodies were purchased from eBioscience and BioLegend: anti-CD45 (30-F11), anti-TCR-β (H57-597), anti-TCRγδ (GL3), anti-CD8α (53–6.7), anti-CD8β (eBioH35-17.2), anti-CD4 (RM4-5), anti-CD103 (2E7), anti-Foxp3 (FJK-16s), anti-T-bet (eBio4B10), anti-IL-17A (TC11-18H10.1), anti-IL-22 (IL22JOP), anti-IFN-γ (XMG1.2), anti-IL-10 (JES5-16E3), anti-IL-9 (RM9a4), anti-IL-4 (11B11), and viability stain kit (Zombie Yellow™ Fixable Viability Kit, BioLegend). Cells were fixed and permeabilized using Foxp3/Transcription Factor Staining Buffer (eBioscience) prior to transcription factor staining and intra-nuclear staining. Intracellular cytokine staining was performed after 3.5 h stimulation using 25 ng/ml of Phorbol 12-myristate 13-acetate (PMA, Sigma-Aldrich), 3 μM of Ionomycin (Sigma-Aldrich), and 1 μg/ml of Brefeldin A (GolgiPlug™ BD Bioscience). Stimulated cells were permeabilized using Cell Fixation/Permeabilization Kits (BD Bioscience) following the instruction from manufactures.

### Statistical Analysis

The unpaired two-tailed Student *t*-test was performed to determine the *p*-values between two groups. The one-way analysis of variance analysis (ANOVA) was used to calculate the significance for comparisons more than two groups using Prism software (GraphPad), except indicated otherwise.

## Results

### TCRβ^+^CD8αα^+^ IELs Reside in Oral Mucosa

**Figure 1:**
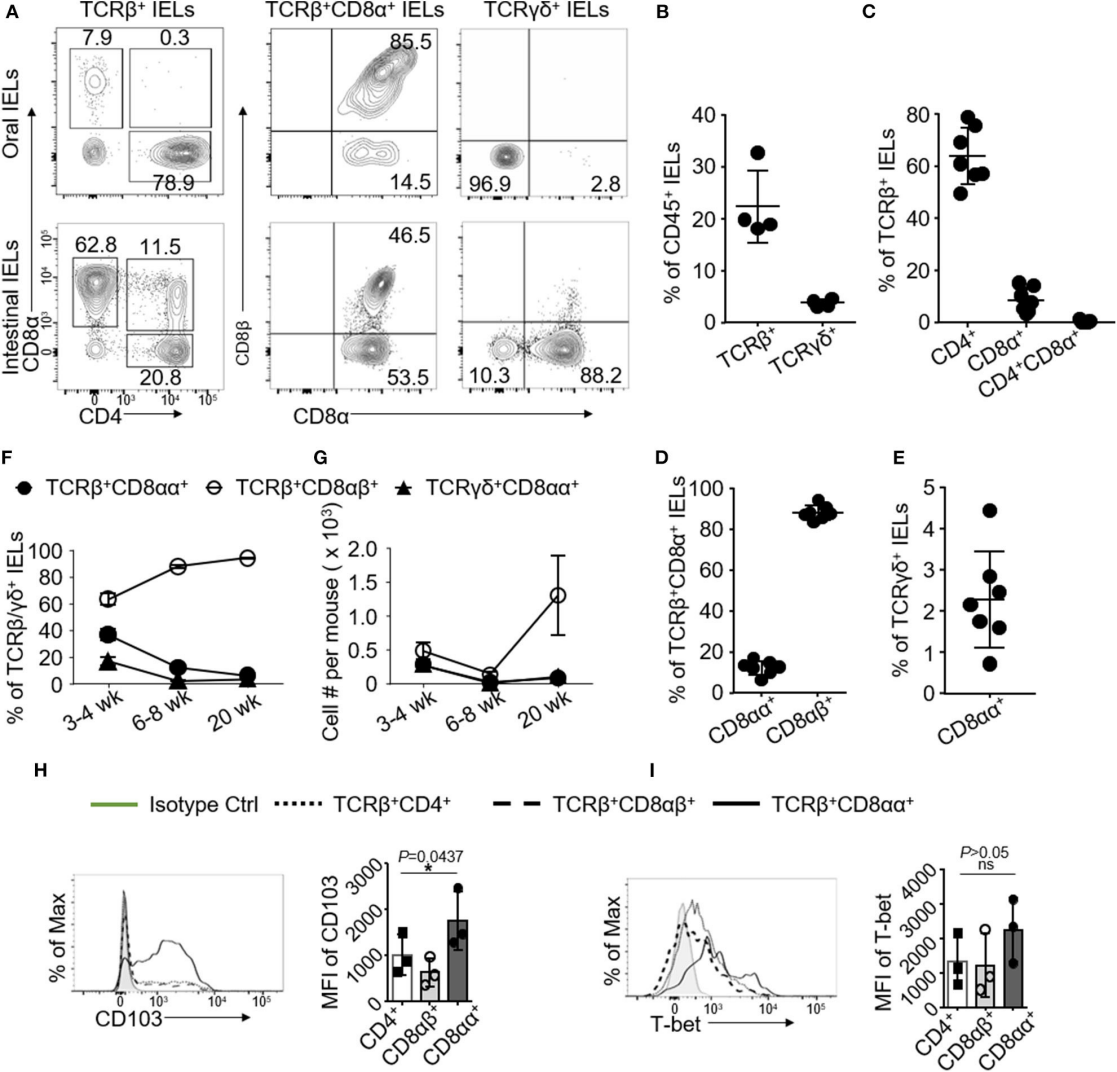
Oral IELs were isolated from 2 to 4 mice and pooled in each experiment. Data are representative for 4–7 independent experiments. **(A)** Representative flow cytometric analysis of oral (upper raw) or intestinal (lower raw) IELs. Cell were gated on CD45^+^ TCRβ^+^CD4^+^, TCRβ^+^CD8^+^, TCRβ^+^CD8αβ^+^, TCRβ^+^CD8αα^+^, and TCRγδ^+^ IELs. **(B)** Frequency of CD45^+^ TCRβ^+^ or TCRγδ^+^ IELs in oral mucosa. **(C)** Frequency of TCRβ^+^CD4^+^, TCRβ^+^CD8α^+^, or TCRβ^+^CD4^+^CD8α^+^ IELs in oral mucosa. **(D)** Frequency of TCRβ^+^CD8αβ^+^ or TCRβ^+^CD8αα^+^ IELs in oral mucosa. **(E)** Frequency of TCRγδ^+^CD8αα^+^ IELs in oral mucosa. **(F)** Frequency and **(G)** total number of TCRβ^+^ or TCRγδ^+^ oral mucosa IEL subtypes at different time points. **(H)** Representative histograms and mean fluorescence intensity (MFI) showing expression of CD103 and **(I)** T-bet among different subsets of oral mucosa IELs. **(H,I)** were determined by one-way ANOVA. (^*^p < 0.05 and ns, not significant) IELs were isolated from oral mucosa and small intestines in 6- to 9-week-old C57BL/6 mice unless stated otherwise **(A–E, H,I)**.

TCRβ^+^CD8αα^+^ IELs in the gut represent in the frontal lining of the mucosal immune system to maintain immune homeostasis (1, 4, 21). To investigate oral IELs, we isolated IELs from oral mucosa using procedures established by our lab and analyzed them using flow cytometry. We first showed that the phenotypes of oral IELs were similar to the gut IELs, but the frequency of oral IELs was lower than those in the gut. We identified ~20% of TCRβ-positive IELs in the gated CD45^+^ immune cells. Compared to the small intestine, there were more CD4^+^ than CD8^+^ IELs (Figures 1A–C). We observed that the CD4^+^CD8^+^ (double positive) TCRβ^+^ IELs that exist in the small intestine (21) were absent in oral mucosa (Figures 1A,C).

Both TCRβ^+^CD8αα^+^ and TCRγδ^+^ IELs are referred to as unconventional IELs, which are developed via a distinct pathway and show unique functions compared to conventional TCRβ^+^CD4^+^ or CD8αβ^+^ T cells (1, 4, 21, 22). As it is unknown whether the TCRβ^+^CD8αα^+^ IELs are located in oral mucosa, we showed that there were about ~15% of TCRβ^+^CD8αα^+^ IELs among the TCRβ^+^CD8^+^ IELs(containing TCRβ^+^CD8αα^+^ and TCRβ^+^CD8αβ^+^ subsets of IELs) in oral mucosa, which were less frequent compared to their counterparts in GI tract (Figures 1A,D). TCRγδ^+^ CD8αα^+^ IELs, which are another subset of unconventional IELs in oral mucosa, also exhibited different phenotypes than their counterparts in GI tract. We have shown before that more than 50% of TCRγδ^+^ IELs from GI tract co-express CD8αα receptors (21); however, in oral mucosa we determined that up to ~90% of TCRγδ^+^ IELs were negative for both CD8 and CD4 molecules (Figures 1A,E). In addition, we also characterized oral LPLs and surprisingly observed that oral LPLs also contained TCRβ^+^CD8αα^+^ T cells that occurred approximately as frequent as the oral IELs (Supplementary Figure 1A). As expected, we observed that CD8αα^+^ T cells were absent in peripheral blood, cervical DLNs and NALT (Supplementary Figure 1B). Interestingly, we found that increased frequency and total number of TCRβ^+^CD8αα^+^ oral IELs were in younger mice (3 to 4-weeks of age) compared to older mice (6 to 8-weeks and 20-weeks) (Figures 1F,G). We also observed higher frequency of TCRβ^+^CD8αα^+^ IELs in the buccal mucosa, gingiva and oral floor compared to tongue mucosa (Supplementary Figure 1C). Importantly, we determined that all oral IEL subsets express CD103, a critical molecule retaining mucosal T cells (23). Intriguingly, the expressions of CD103 and T-bet in TCRβ^+^CD8αα^+^ IELs were higher than that in TCRβ^+^CD8αβ^+^ and TCRβ^+^CD4^+^ IELs (Figures 1H,I). Altogether, we have uncovered that CD8αα^+^ IELs reside in oral mucosal tissues.

### TCRβ^+^CD8αα^+^ IELs Are Regulated by TGF-β in the Oral Mucosa

**Figure 2:**
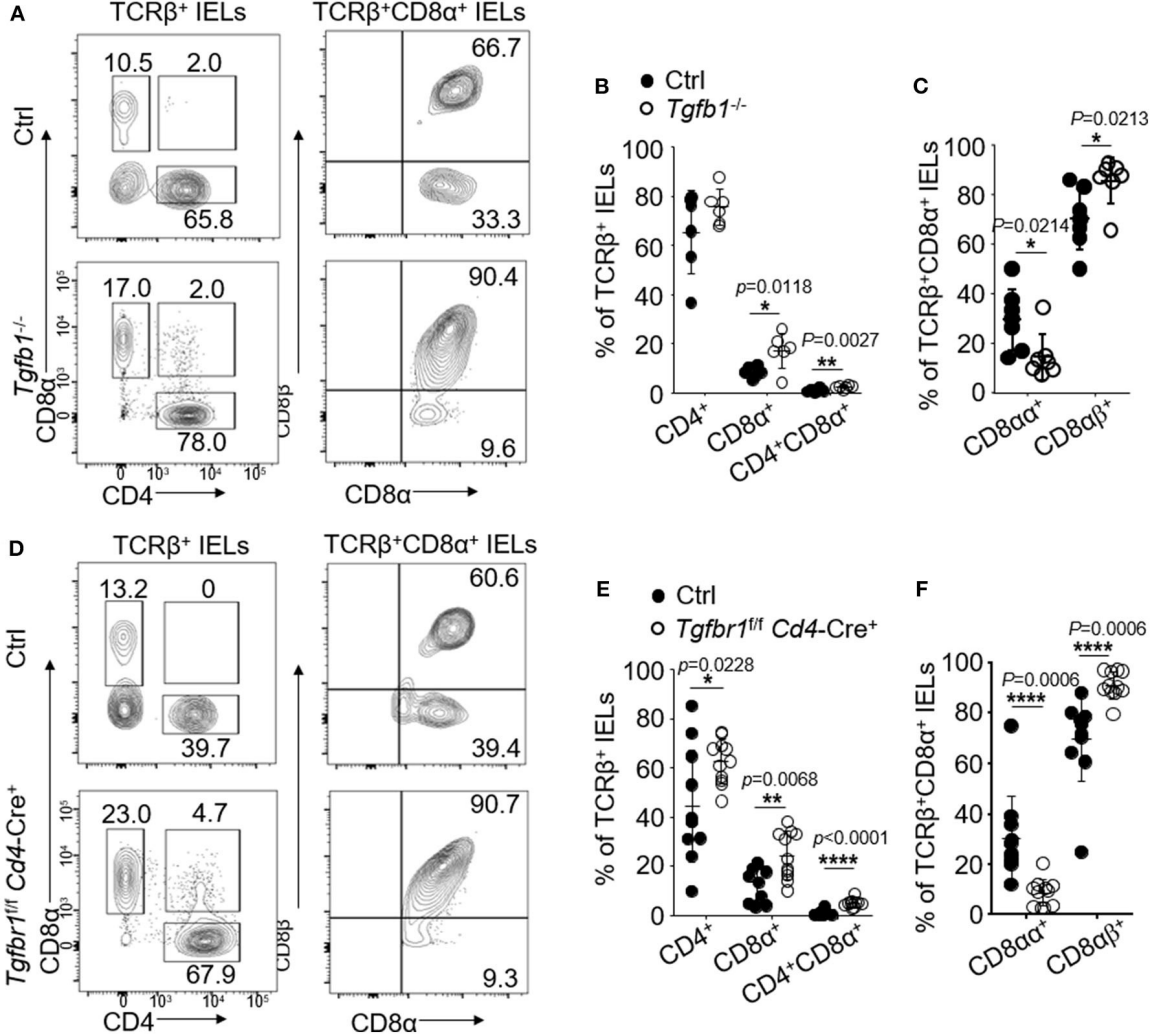
IELs were isolated from 2 to 4 mice and pooled in each experiment. Flow cytometric data are representative for three independent experiments. **(A)** Representative plots of TCRβ^+^CD4^+^, TCRβ^+^CD8α^+^, TCRβ^+^CD8αβ^+^, or TCRβ^+^CD8αα^+^ oral IELs isolated from 2- to 3-week-old *Tgfb1*^−/−^ mice and age matched littermate controls (*Tgfb1*^+/+^). **(B)** Frequency of TCRβ^+^CD4^+^, TCRβ^+^CD8α^+^, and TCRβ^+^CD4^+^CD8α^+^ oral IELs. Significance was determined using unpaired two-tailed Student's *t*-test (^*^p < 0.05 and ^**^p < 0.01). **(C)** Frequency of TCRβ^+^CD8αα^+^ and TCRβ^+^CD8αβ^+^ IELs. Cells were gated on TCRβ^+^CD8α^+^. Significance was determined using unpaired two-tailed Student's *t*-test (^*^p < 0.05). **(D)** Representative plots of TCRβ^+^CD4^+^, TCRβ^+^CD8α^+^, and TCRβ^+^CD8αβ^+^ or TCRβ^+^CD8αα^+^ oral IELs isolated from 2- to 4-week-old *Tgfbr1*^*f*/*f*^ *Cd4*-Cre^+^ mice and age matched littermate controls (*Tgfbr1*^+/+^ *Cd4*-Cre^+^ or *Cd4*-Cre^−^). **(E)** Frequency of TCRβ^+^CD4^+^, TCRβ^+^CD8α^+^, and TCRβ^+^CD4^+^CD8α^+^ oral IELs. Significance was determined using unpaired two-tailed Student's *t*-test (^*^p < 0.05, ^**^p < 0.01, and ^****^p < 0.0001). **(F)** Frequency of TCRβ^+^CD8αα^+^ and TCRβ^+^CD8αβ^+^ oral IELs. Significance was determined using unpaired two-tailed Student's *t*-test (^****^p < 0.0001).

We have previously shown that TGF-β controls the development of TCRβ^+^CD8αα^+^ IELs in GI tract (21). We next studied whether oral TCRβ^+^CD8αα^+^ IELs were affected by TGF-β signaling. We examined TCRβ^+^CD8αα^+^ IELs in the mice (*Tgfbr1*-floxed *Cd4*-Cre mice) in which T cells were specifically ablated of TGF-β receptor I (Figures 2D–F) and in the TGF-β1 null (*Tgfb1* KO) mice (Figures 2A–C). We observed substantially increased frequency of TCRβ^+^CD4^+^ in *Tgfbr1*-floxed *Cd4*-Cre mice and total TCRβ^+^CD8^+^ IELs in TGF-β1 null and *Tgfbr1*-floxed *Cd4*-Cre mice (Figures 2A–F). Despite the increase of TCRβ^+^CD8^+^ IELs, however, we observed a significant reduction in frequency of TCRβ^+^CD8αα^+^ IELs (Figures 2C,F), as well as a reduction of CD8α mean fluorescence intensities (Supplementary Figure 2D), which was consistent with the findings in the GI tract (21). We also found that in *Tgfb1* KO and *Tgfbr1*-floxed *Cd4*-Cre^+^ mice, TCRβ^+^CD4^+^CD8α^+^ IELs were significantly increased (Figures 2A,B,D,E) compared to their counterparts in the GI track. Surprisingly, the TCRβ^+^CD4^+^ CD8α^+^ IELs were absent in controls. Unexpectedly, most TCRβ^+^CD4^+^ CD8α^+^ IELs in the TGF-β signaling knockout mice were CD8αβ-positive (Supplementary Figure 2C). Moreover, we did not find significant difference of TCRγδ^+^CD8αα^+^IELs between knockout and control mice (Supplementary Figures 2A,B). In contrast to TGF-β deficient mice, mice with overexpression of TGF-β1 (*Alb/TGF*-β*1*) showed increase in TCRβ^+^CD8α^+^ and TCRβ^+^CD4^+^CD8^+^ IELs in the oral mucosa. Importantly, we determined a significant increase in the TCRβ^+^CD8αα^+^ oral IELs from TGF-β1 transgenic mice compared to controls (Supplementary Figure 2E). These data altogether indicate that TGF-β regulates TCRβ^+^CD8αα^+^ IELs in oral mucosa.

### IFN-γ Production in Oral TCRβ^+^CD8αα^+^ IELs Is Enhanced in the Absence of TGF-β

**Figure 3:**
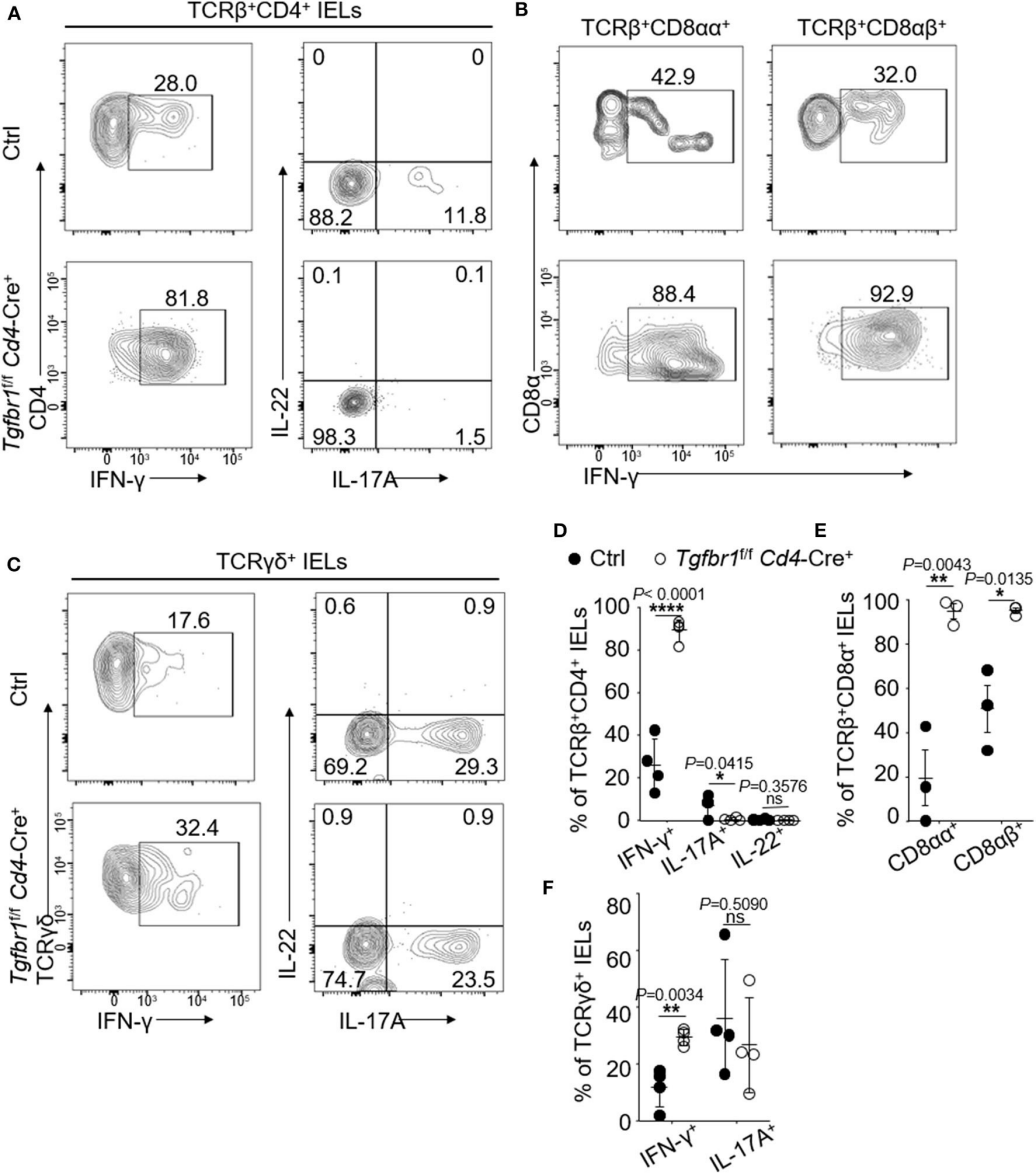
Oral IELs were isolated from 2- to 4-week-old *Tgfbr1*^*f*/*f*^ *Cd4*-Cre^+^ mice and age matched controls (*Tgfbr1*^+/+^ *Cd4*-Cre^+^ or *Cd4*-Cre^−^). Each dot in dot plots is representing individual experiment in which cells were isolated from two mice and pooled. Flow cytometric plots are representative of 2–3 independent experiments. **(A)** Representative plots of TCRβ^+^CD4^+^IFN-γ+, TCRβ^+^CD4^+^IL-17A^+^, and TCRβ^+^CD4^+^IL-22^+^ oral IELs. **(B)** Representative plots of TCRβ^+^CD8αα^+^IFN-γ+ or TCRβ^+^CD8αβ^+^IFN-γ^+^ oral IELs. **(C)** Representative plots of TCRγδ^+^ IFN-γ^+^, TCRγδ^+^IL-17A^+^, and TCRβ^+^IL-22^+^ oral IELs. **(D)** Summarized data showing frequency of cytokines produced by TCRβ^+^CD4^+^ oral IELs. **(E)** Summarized data showing frequency of cytokines produced by TCRβ^+^CD8αα^+^ or TCRβ^+^CD8αβ^+^ oral IELs. **(F)** Summarized data showing frequency of cytokines produced by TCRγδ^+^ oral IELs. Significance **(D–F)** was determined by unpaired two-tailed Student's *t*-test (^*^p < 0.05, ^**^p < 0.01, ^****^p < 0.0001, and ns, not significant).

Gut IELs have been known to contribute to the integrity of immune responses to microorganisms (4). To investigate the functions of oral IELs in physiological conditions, we assessed the cytokine production profiles of each oral IEL subset of normal wild type mice. In the steady conditions, the TCRβ^+^CD4^+^ oral IELs mainly produced IFN-γ, IL-17A, IL-4, and IL-10, but rarely produces IL-9, IL-22, and IL-13 (Supplementary Figures 3A,B). We also observed that a large population of Foxp3^+^ regulatory T (Treg) cells were contained in TCRβ^+^CD4^+^ IELs (Supplementary Figures 3A,B). In contrast, oral TCRβ^+^CD8αα^+^ IELs produced IFN-γ but not IL-17A, IL-4, IL-9, IL-22, or IL-10 (Supplementary Figures 3C,D). In addition, we found there was no difference of the cytokine profile between oral TCRβ^+^CD8αα^+^ and TCRβ^+^CD8αβ^+^ IELs (Supplementary Figures 3C,D). For unconventional TCRγδ^+^ IELs, we observed IFN-γ, IL-17A, IL-4, and IL-10 were the main cytokines, but not IL-9 and IL-22 (Supplementary Figures 3E,F). In *Tgfbr1*-floxed *Cd4*-Cre^+^ mice, both conventional CD4^+^ and CD8αα^+^ T cells and unconventional TCRβ^+^CD8αα^+^ and TCRγδ^+^CD8α^+^ IELs produced larger amounts of IFN-γ than control mice (Figures 3A–F). TCRβ^+^CD4^+^ IELs in the knockout mice however produced less IL-17 compared to controls.

### IMQ Treatment Increases Frequency of TCRβ^+^CD8αα^+^ IELs and Their IFN-γ Production

**Figure 4:**
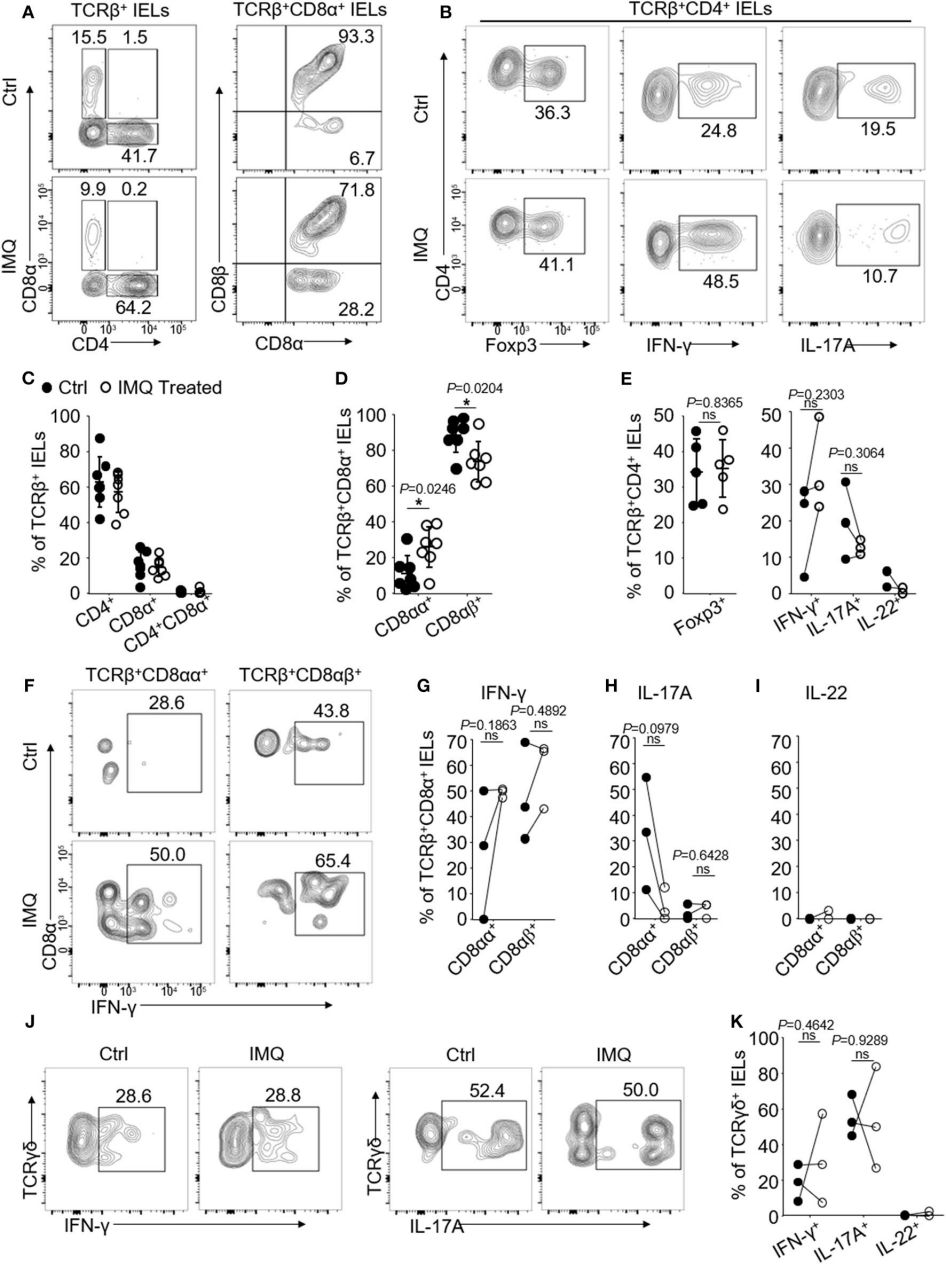
Oral IELs were isolated from mice received IMQ or pure petroleum jelly treatment (Controls, Ctrl). Each dot in dot plots represents individual experiment in which cells were isolated from two mice and pooled. Flow cytometric plots are representatives of 2–3 independent experiments. **(A)** Representative plots of TCRβ^+^CD4^+^, TCRβ^+^CD8α^+^, TCRβ^+^CD8αβ^+^, or TCRβ^+^CD8αα^+^ oral IELs in Control or IMQ-treated mice. **(B)** Representative plots of TCRβ^+^CD4^+^Foxp3^+^ Tregs, TCRβ^+^CD4^+^IFN-γ^+^, or TCRβ^+^CD4^+^IL-17A^+^ oral IELs. **(C)** Summarized data showing frequency of TCRβ^+^CD4^+^, TCRβ^+^CD8α^+^, or TCRβ^+^CD4^+^CD8α^+^ oral IELs in Control or IMQ-treated mice. **(D)** Summarized data showing frequency of TCRβ^+^CD8αβ^+^ or TCRβ^+^CD8αα^+^ oral IELs in Control or IMQ-treated mice. **(E)** Summarized data showing frequency of cytokines produced by TCRβ^+^CD4^+^ oral IELs in control and IMQ-treated mice. **(F)** Representative plots of TCRβ^+^CD8αβ^+^IFN-γ^+^ or TCRβ^+^CD8αα^+^IFN-γ^+^ oral IELs in Control and IMQ-treated mice. **(G)** Summarized data showing frequency of TCRβ^+^CD8αβ^+^IFN-γ^+^ or TCRβ^+^CD8αα^+^IFN-γ^+^ oral IELs in control or IMQ-treated mice. **(H)** Summarized data showing frequency of TCRβ^+^CD8αβ^+^IL-17A^+^ or TCRβ^+^CD8αα^+^IL-17A^+^ oral IELs in control or IMQ-treated mice. **(I)** Summarized data showing frequency of TCRβ^+^CD8αβ^+^IL-22^+^ or TCRβ^+^CD8αα^+^IL-22^+^ oral IELs in control or IMQ-treated mice. **(J)** Representative plots of TCRγδ^+^IFN-γ^+^ or TCRγδ^+^IL-17A^+^ IELs in control and IMQ-treated mice. **(K)** Summarized data showing frequency of cytokines produced by TCRγδ^+^ oral IELs in control and IMQ-treated mice. Significance **(E,G,H,K)** was determined using unpaired two-tailed Student's *t*-test (ns, not significant and ^*^P < 0.05).

We have shown that IMQ-treated mice developed psoriasis and systemic inflammation following 6-day treatments (18). We next asked whether oral TCRβ^+^CD8αα^+^ IELs could be influenced by systemic inflammation induced by IMQ treatment. We observed that the IMQ-treated mice developed skin lesions and inflammation as reported (18), however, neither significant lesions nor pathological changes were observed in the buccal and tongue mucosa (Supplementary Figure 4B). We isolated and assessed oral IELs and found a significant increase in the frequency of unconventional TCRβ^+^CD8αα^+^ but a decrease in TCRβ^+^CD8αβ^+^ IELs in the IMQ-treated mice (Figures 4A,D). Total CD8^+^ IEL frequency was not significantly changed compared to control mice (Figures 4A,C,D). Importantly, we observed that oral TCRβ^+^CD8αα^+^ IELs enhanced IFN-γ production after IMQ treatment (Figures 4F,G), and no significant difference in IL-17A and IL-22 production (Figures 4H,I). In the CD4^+^ IELs, we observed an elevation of IFN-γ and a decrease of IL-17A and IL-22 production, but no significant difference in Treg cells (Figures 4B,E). However, the IMQ treatment had no significant effect on the frequency of TCRγδ^+^CD8αα^+^ IELs and their cytokine production (Figures 4J,K and Supplementary Figure 4C). The data suggest that the IMQ-triggered inflammation affects TCRβ^+^CD8αα^+^ numbers and function.

### Neonatal Antibiotic Treatment Decreases TCRβ^+^CD8αα^+^ IELs

**Figure 5:**
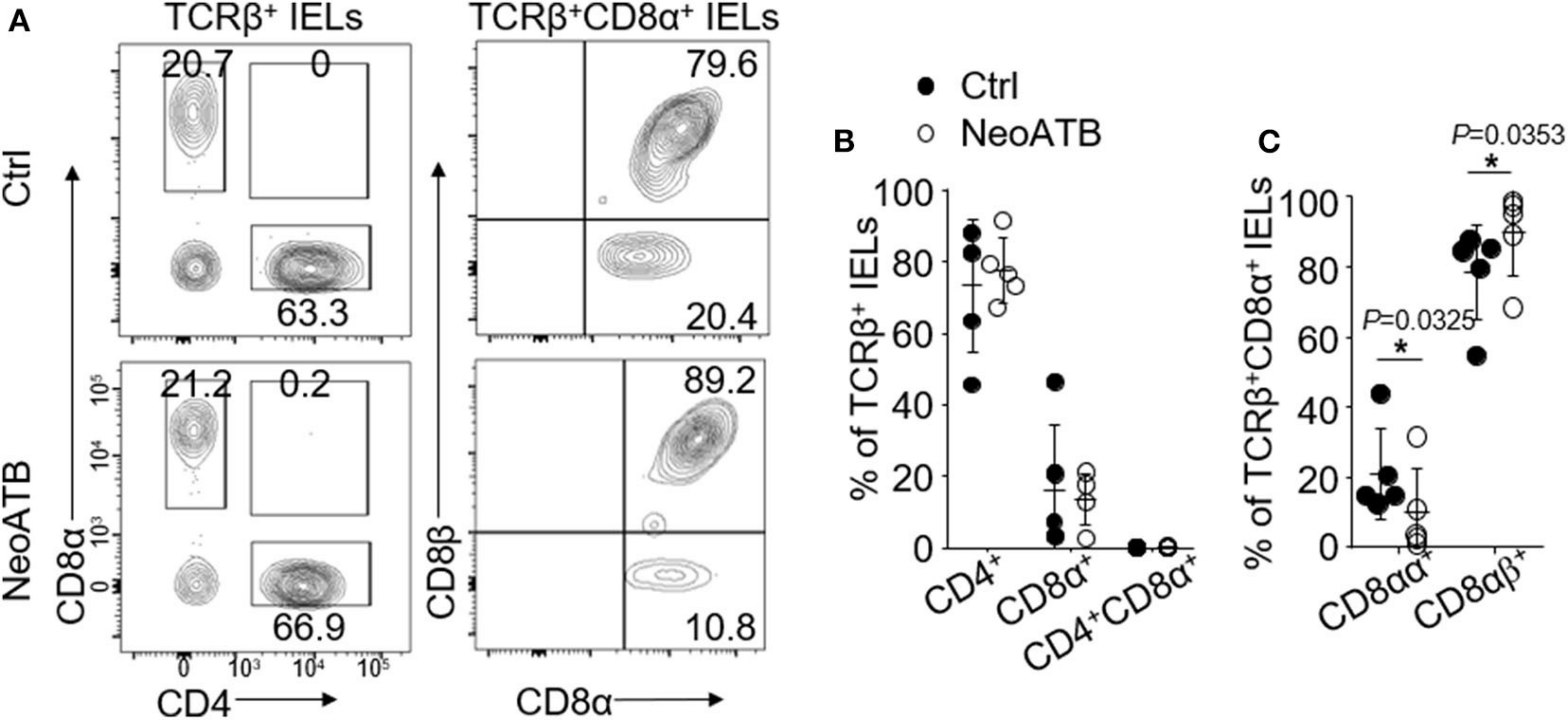
Oral IELs were isolated from mice that received neonatal antibiotic treatment in first 3 weeks of their life and after weaning were housed in normal environment for next 8 weeks (NeoATB), or controls (Ctrl). Each dot in dot plots is representing individual experiment in which cells were isolated from two mice and pooled. Flow cytometric plots are representative of 2–3 independent experiments. **(A)** Representative plots of TCRβ^+^CD4^+^, TCRβ^+^CD8α^+^, TCRβ^+^CD8αβ^+^, or TCRβ^+^CD8αα^+^ oral IELs in Control and NeoATB mice. **(B)** Summarized data showing frequency of TCRβ^+^CD4^+^, TCRβ^+^CD8α^+^, or TCRβ^+^CD4^+^CD8α^+^ oral IELs in Control and NeoATB mice. **(C)** Summarized data showing frequency of TCRβ^+^CD8αβ^+^ or TCRβ^+^CD8αα^+^ oral IELs in Control and NeoATB mice. Significance was determined using unpaired two-tailed Student's *t*-test (^*^p < 0.05).

It has been shown that neonatal antibiotic treatment causes dysbiosis of microbiota and increased the sensitivity to skin inflammation at the adult age in mice (18). We next studied whether antibiotics treatment in neonatal age (age 0–21 days) would affect the number and function of oral TCRβ^+^CD8αα^+^ IELs. For this, we treated C57BL/6J mice with vancomycin and polymyxin B via drinking water from the date of birth for 3 weeks (Supplementary Figure 4A). The mice were then given regular water and housed for the next additional 8 weeks in a normal environment. We then analyzed the oral IELs and found a significant decrease in the TCRβ^+^CD8αα^+^ IELs and a significant increase in TCRβ^+^CD8αβ^+^ IELs (Figures 5A–C). In contrast, we did not find significant changes of TCRγδ^+^CD8αα^+^ IELs between neonatal antibiotic treated and control mice (Supplementary Figure 4D). The data reveal that dysbiosis induced by neonatal antibiotic treatment results in alterations of TCRβ^+^CD8α^+^ IELs.

## Discussion

TCRβ^+^CD8αα^+^ IELs have been described as unconventional T cells residing particularly within intraepithelial compartments of gut mucosae (1, 4). Previous studies have demonstrated that TGF-β controls intestinal TCRβ^+^CD8αα^+^ IEL development and differentiation (21). However, the phenotype and function of TCRβ^+^CD8αα^+^ IELs in oral mucosa remain unclear. In this report, we have established efficient procedures for isolating IELs and LPLs from oral mucosa and have profiled murine oral IELs by flow cytometry. We have shown that oral IELs share similar phenotypes with GI IELs in terms of their surface markers, as well as in their expression of transcription factors. However, we identified TCRβ^+^CD8αα^+^ IELs exist in normal oral mucosal compartment but are absent in other lymphoid tissues in the oral cavity. We observed that in oral mucosa, the TCRβ^+^CD8αα^+^ maintained higher CD103 and T-bet expression compared to other TCRβ^+^ subsets. This is consistent with recent reports which indicate that gut IELs maintain high expression of CD103 and T-bet (24, 25), which are responsible for their infiltration and trafficking into mucosa, differentiation and clonal expansion *in situ*. Furthermore, we revealed that older mice have fewer TCRβ^+^CD8αα^+^ IELs, which implies that the older the mice are, the more antigens are encountered in the mucosa, promoting infiltration and proliferation of TCRβ^+^CD4^+^ and CD8αβ^+^ subsets in IELs.

We characterized the cytokine profiles of each oral IEL subset and found that under physiological conditions, the conventional TCRβ^+^CD4^+^ subset produce pro-inflammatory cytokine IFN-γ, IL-17A, and IL-4, as well as the anti-inflammatory cytokine IL-10. TCRβ^+^CD8αβ^+^ IELs mainly produce IFN-γ. Interestingly, we found that there were increased numbers of Treg cells in the oral mucosa, indicating a need for more Treg cells in maintaining immune regulation and tolerance in the oral mucosa (5, 26, 27). It has been shown that TCRβ^+^CD8αα^+^ IELs in the genital dermal-epidermal junction exert immune surveillance and immune defense against HSV2 infection via upregulating IFN-γ (28). Here we show that the unconventional TCRβ^+^CD8αα^+^ oral IELs produce dominant IFN-γ rather than other cytokines, and that TCRγδ^+^ IELs produce both IFN-γ and IL-17A. Based on their cytokine profile, the dominant role predicted for unconventional oral IELs would be the protection of oral mucosa from pathogens and microbes under steady conditions. Yet the host defense function of oral CD8αα^+^ IELs in oral disease remains to be studied.

The properties and functions of IELs vary under their diverse cytokine conditions, which in turn affects their cytokine profiles (1, 3). Here we observed that both unconventional and conventional oral IELs significantly upregulate IFN-γ expression in conditional TGF-β receptor knockout mice. We believe that the increased production of IFN-γ was largely due to the decrease in the frequency of Treg cells rather than the defect of Treg cell suppressive function *per se* in the absence of TGF-β signaling, because we showed before (23) that TGF-β receptor I knockout Treg cells exhibited stronger suppression to IFN-γ production in T cells *in vitro* cultures. In addition to Treg cells, the direct suppressive function of TGF-β signaling in T cells may also play a role. Our data indicate that TGF-β is crucial for oral IELs to maintain normal function. The data further supports our previous finding that TGF-β is critical for the development of mucosal TCRβ^+^CD8αα^+^ IELs in the gut (21). Using IMQ-treated mice as a model of systemic inflammation (18), we observed that systemic inflammation at least induced by IMQ drives more oral TCRβ^+^CD8αα^+^ IELs, suggesting these population of oral IELs are important responder to inflammation, although that their function is stimulatory or inhibitory remains to be known. One of the salient features for almost all oral IEL subsets is their high levels of IFN-γ production, which is not seen in other pro-inflammatory cytokine expression, such as IL-17A. Recent studies have suggested that IFN-γ might be responsible for the expansion of peripheral TCRβ^+^CD8αα^+^ IELs (24, 25, 29). It would be interesting to study whether IFN-γ also plays a similar role for TCRβ^+^CD8αα^+^ IELs in oral cavity. In our previous studies by using IMQ to induce skin psoriasis in mice (18) we observed not only the locations that applied the medication showed pathological lesions but also some inflammation in GI tract and spleen suggesting a systemic inflammation. Intriguingly, we noted that the oral mucosa from IMQ-treated mice exhibited no apparent mucosal lesions. In addition, our data showed that IMQ treatments drove more CD8αα^+^ oral IELs but no changes in Treg cells. This suggests that CD8αα^+^ IELs might possess an immune regulatory function, which may account for the lack of lesions of the oral mucosa by suppressing infiltration of the systemic inflammatory cells, a fascinating question to study in the future.

Lastly but importantly, as we reported before (18), early neonatal antibiotics treatment results in changes and dysbiosis of the gut microbiota in adult life. In the neonatal antibiotics treatment model, we observed that the alteration of the microbiota was associated with significant decrease of CD8αα^+^ oral IELs, providing us a starting point to understand the relationship and mutual regulation between microbiota and oral TCRβ^+^CD8αα^+^ IELs in maintaining delicate balance of oral IELs and immune responses in oral mucosa.

## Data Availability Statement

All datasets generated for this study are included in the article/Supplementary Material.

## Ethics Statement

Animal studies were approved by the Animal Care and Use Committees of National Institute of Dental and Craniofacial Research and by the NIH, and conducted according to guidelines of the NIH for use and care of live animals.

## Author's Note

All animals were bred and maintained in animal facilities of Veterinary Resources Core at NIDCR, NIH. Flow cytometry and microscopy were performed in the Combined Technical Research Core of NIDCR, NIH.

## Author Contributions

RW and PZ designed and performed the experiments, interpreted the data, and drafted the paper. DZ and WJ performed the experiments and analyzed the data. HW provided critical scientific input and helped experiments. WC conceived of and supervised all studies, designed the experiments, and edited the manuscript. All authors contributed to the article and approved the submitted version.

## Conflict of Interest

The authors declare that the research was conducted in the absence of any commercial or financial relationships that could be construed as a potential conflict of interest.

## Supplementary Material

Supplementary Figure 1 **(A)** Representative flow cytometric plots of TCRβ^+^CD4^+^, TCRβ^+^CD8α^+^, TCRβ^+^CD8αα^+^, TCRβ^+^CD8αβ^+^, or TCRγδ^+^ lamina propria lymphocytes (LPLs) in oral mucosa. Lower raw, summarized data showing frequency of CD45^+^ TCRβ^+^ or TCRγδ^+^ LPLs, TCRβ^+^CD4^+^, TCRβ^+^CD8α^+^, TCRβ^+^CD4^+^CD8α^+^, TCRβ^+^CD8αβ^+^, TCRβ^+^CD8αα^+^, and TCRγδ^+^CD8αα^+^ oral mucosa LPLs. **(B)** Representative plots of TCRβ^+^CD4^+^, TCRβ^+^CD8α^+^, TCRβ^+^CD8αα^+^, TCRβ^+^CD8αβ^+^, or TCRγδ^+^ cells from peripheral blood, cervical draining lymph nodes (Cervical DLN) and NATL. **(C)** Representative plots of TCRβ^+^CD4^+^, TCRβ^+^CD8α^+^, TCRβγ^+^CD8αα^+^, TCRβ^+^CD8αβ^+^, or TCRγδ^+^ from tongue mucosa (Tongue) and buccal, oral floor, and gingival mucosa (mucosa). Summarized data showing frequency of TCRβ^+^CD4^+^, TCRβ^+^CD8α^+^, TCRβ^+^CD8αα^+^, TCRβ^+^CD8αβ^+^, or TCRγδ^+^ IELs of buccal or tongue mucosa. LPLs and IELs were isolated from oral mucosa or small intestines in 6- to 9-week-old C57BL/6 mice. Oral IELs were isolated from 2 to 4 mice and pooled in each experiment. Data are representative of 2 and 3 independent experiments.

Supplementary Figure 2 **(A)** Representative flow cytometric plots of TCRγδ^+^CD8αα^+^ oral IELs from *Tgfb1*^−/−^ mice and age matched littermate controls (*Tgfb1*^+/+^). **(B)** Representative plots of TCRγδ^+^CD8αα^+^ oral IELs from *Tgfbr1*^*f*/*f*^ CD4-cre^+^ mice and age matched littermate controls. **(C)** Representative plots of TCRβ^+^CD8αα^+^ vs. TCRβ^+^CD8β^+^ gated on TCRβ^+^CD4^+^CD8α^+^ oral IELs from *Tgfbr1*^+/+^ *Cd4*-Cre^+^ mice. **(D)** CD8α expression of TCRβ^+^ oral IELs from 2- to 4-week-old *Tgfbr1*^*f*/*f*^ *Cd4*-Cre^+^ mice and age matched controls (*Tgfbr1*^+/+^ *Cd4*-Cre^+^ or *Cd4*-Cre^−^). **(E)** Representative plots of TCRβ^+^CD4+, TCRβ^+^CD8αα^+^, TCRβ^+^CD8β^+^, and TCRγδ^+^CD8αα^+^ oral IELs from *Alb/Tgfb1* transgenic mice and age-matched controls.

Supplementary Figure 3 IELs were isolated from 6- to 8-week-old C57BL/6 mice. Each dot in dot plots is representing individual experiment in which cells were isolated from two mice and pooled. Flow cytometric plots are representative of 4–7 independent experiments. **(A)** Representative plots of Foxp3, IFN-γ, IL-17A, IL-4, and IL-10 in TCRβ^+^CD4^+^ oral IELs. **(B)** Summarized data showing frequency of cytokine production from oral IELs. **(C)** Representative plots of TCRβ^+^CD8αα^+^IFN-γ^+^ or TCRβ^+^CD8αα^+^IFN-γ^+^ oral IELs. **(D)** Summarized data showing frequency of cytokine production from TCRβ^+^CD8αα^+^ or TCRβ^+^CD8αβ^+^ oral IELs. **(E)** Representative plots of TCRγδ^+^IFN-γ^+^ or TCRγδ^+^IL-17A^+^ oral IELs. **(F)** Summarized data showing frequency of cytokine production from TCRγδ^+^ oral IELs. **(G)** Cell number of IFN-γ^+^ IELs of TCRβ^+^CD8αα^+^ or TCRβ^+^CD8αβ^+^ subsets from *Tgfbr1*^*f*/*f*^ *Cd4*-Cre^+^ mice and age matched littermate controls.

Supplementary Figure 4 **(A)** Immunization scheme of neonatal antibiotic treatment. **(B)** H&E stained buccal and tongue mucosa sections from Control or IMQ treated mice. **(C)** Representative flow cytometric plots of TCRγδ^+^CD8αα^+^ oral IELs from Control or IMQ treated mice. **(D)** Representative plots of TCRγδ^+^CD8αα^+^ oral IELs from Control or NeoATB mice.
