# Supplementary figures and images for: Identification and Regulation of TCRαβ+CD8αα+ Intraepithelial Lymphocytes in Murine Oral Mucosa
Source: Front Immunol. 2020 Aug 4;11:1702. doi: 10.3389/fimmu.2020.01702 (PMC7417446; doi:10.3389/fimmu.2020.01702)

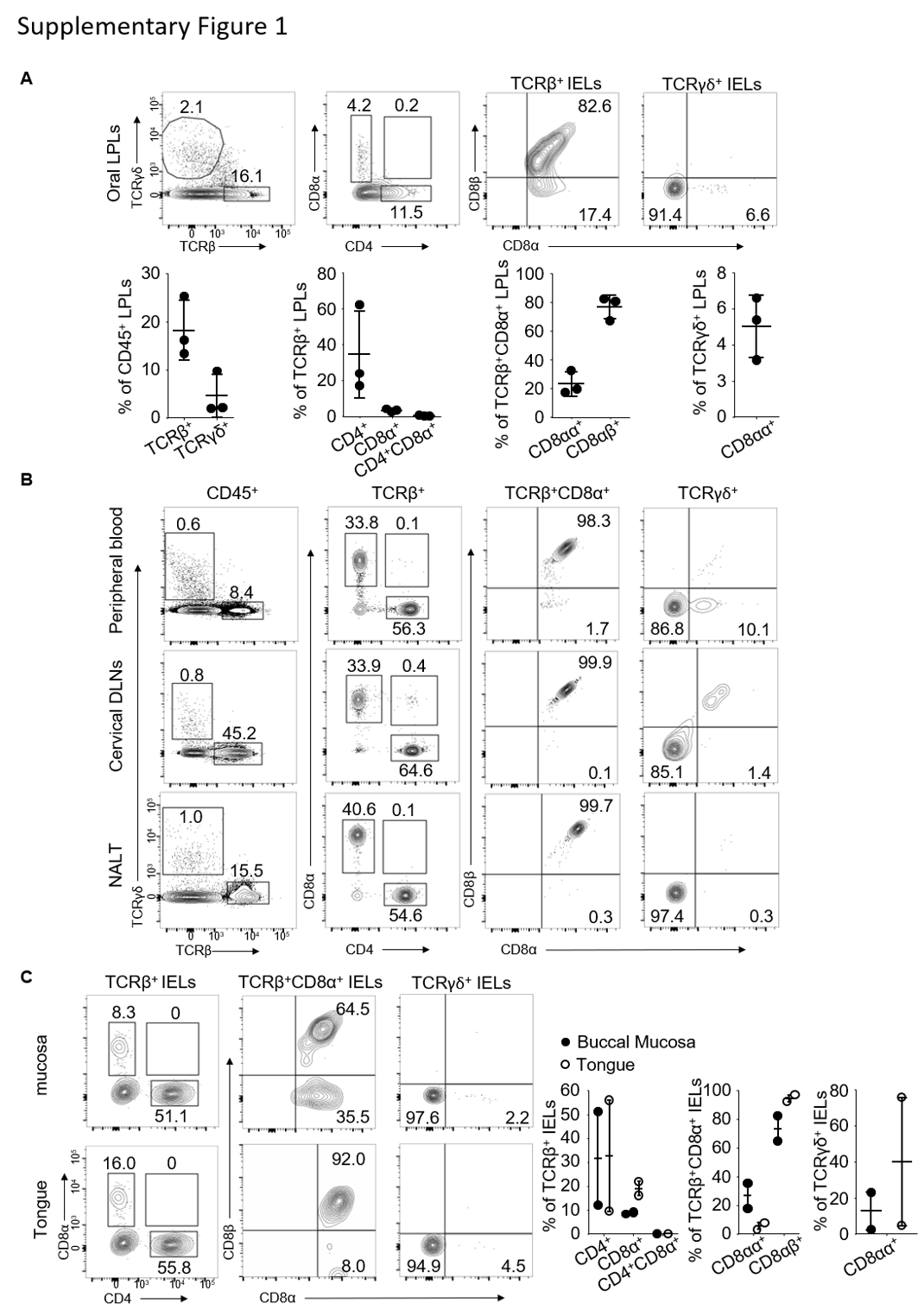

Supplement: Supplementary Figure 1 — (A) Representative flow cytometric plots of TCRβ+CD4+, TCRβ+CD8α+, TCRβ+CD8αα+, TCRβ+CD8αβ+, or TCRγδ+ lamina propria lymphocytes (LPLs) in oral mucosa. Lower raw, summarized data showing frequency of CD45+ TCRβ+ or TCRγδ+ LPLs, TCRβ+CD4+, TCRβ+CD8α+, TCRβ+CD4+CD8α+, TCRβ+CD8αβ+, TCRβ+CD8αα+, and TCRγδ+CD8αα+ oral mucosa LPLs. (B) Representative plots of TCRβ+CD4+, TCRβ+CD8α+, TCRβ+CD8αα+, TCRβ+CD8αβ+, or TCRγδ+ cells from peripheral blood, cervical draining lymph nodes (Cervical DLN) and NATL. (C) Representative plots of TCRβ+CD4+, TCRβ+CD8α+, TCRβγ+CD8αα+, TCRβ+CD8αβ+, or TCRγδ+ from tongue mucosa (Tongue) and buccal, oral floor, and gingival mucosa (mucosa). Summarized data showing frequency of TCRβ+CD4+, TCRβ+CD8α+, TCRβ+CD8αα+, TCRβ+CD8αβ+, or TCRγδ+ IELs of buccal or tongue mucosa. LPLs and IELs were isolated from oral mucosa or small intestines in 6- to 9-week-old C57BL/6 mice. Oral IELs were isolated from 2 to 4 mice and pooled in each experiment. Data are representative of 2 and 3 independent experiments. [file Image_1.TIF]

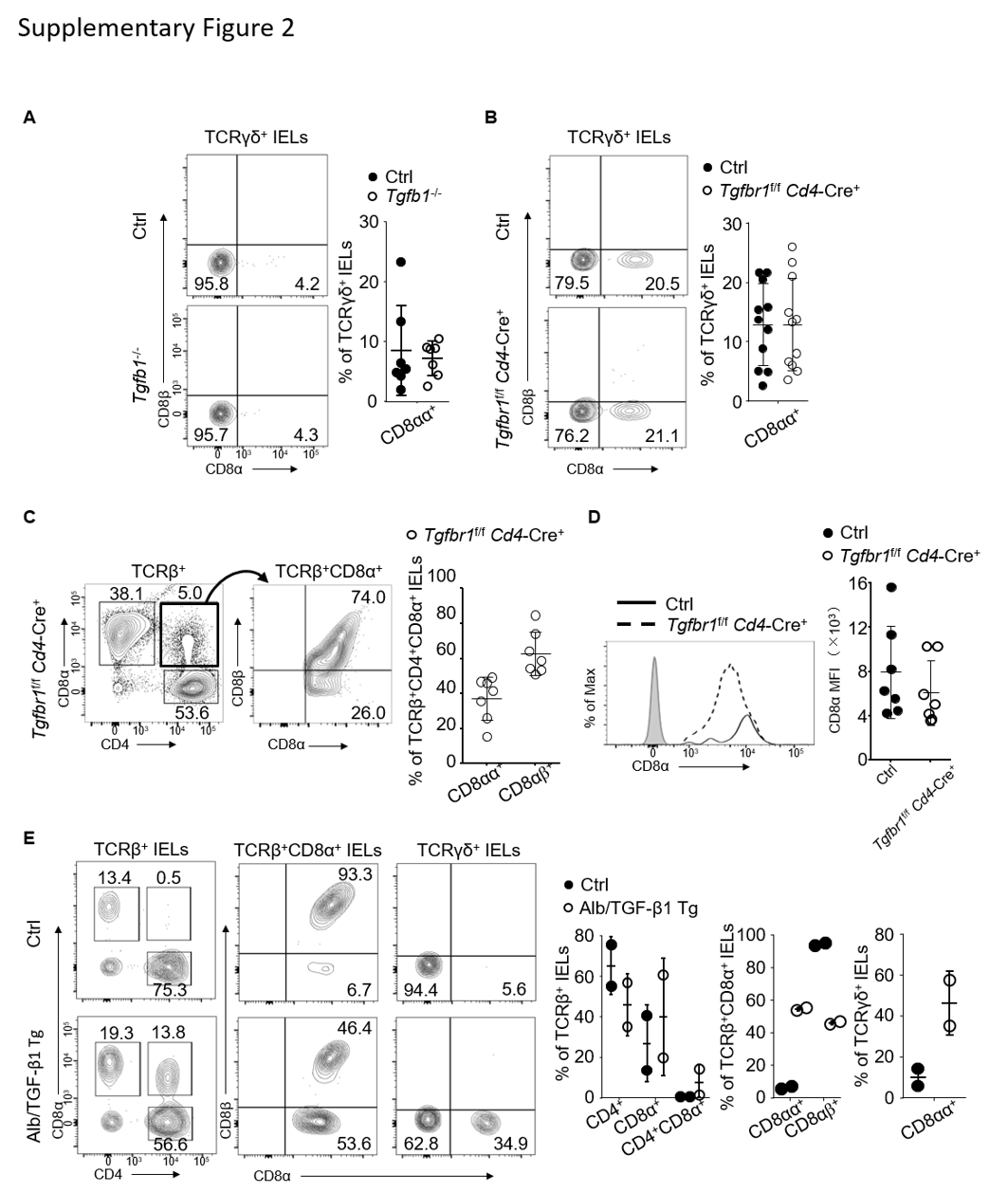

Supplement: Supplementary Figure 2 — (A) Representative flow cytometric plots of TCRγδ+CD8αα+ oral IELs from Tgfb1−/− mice and age matched littermate controls (Tgfb1+/+). (B) Representative plots of TCRγδ+CD8αα+ oral IELs from Tgfbr1f/f CD4-cre+ mice and age matched littermate controls. (C) Representative plots of TCRβ+CD8αα+ vs. TCRβ+CD8β+ gated on TCRβ+CD4+CD8α+ oral IELs from Tgfbr1+/+ Cd4-Cre+ mice. (D) CD8α expression of TCRβ+ oral IELs from 2- to 4-week-old Tgfbr1f/f Cd4-Cre+ mice and age matched controls (Tgfbr1+/+ Cd4-Cre+ or Cd4-Cre−). (E) Representative plots of TCRβ+CD4+, TCRβ+CD8αα+, TCRβ+CD8β+, and TCRγδ+CD8αα+ oral IELs from Alb/Tgfb1 transgenic mice and age-matched controls. [file Image_2.TIF]

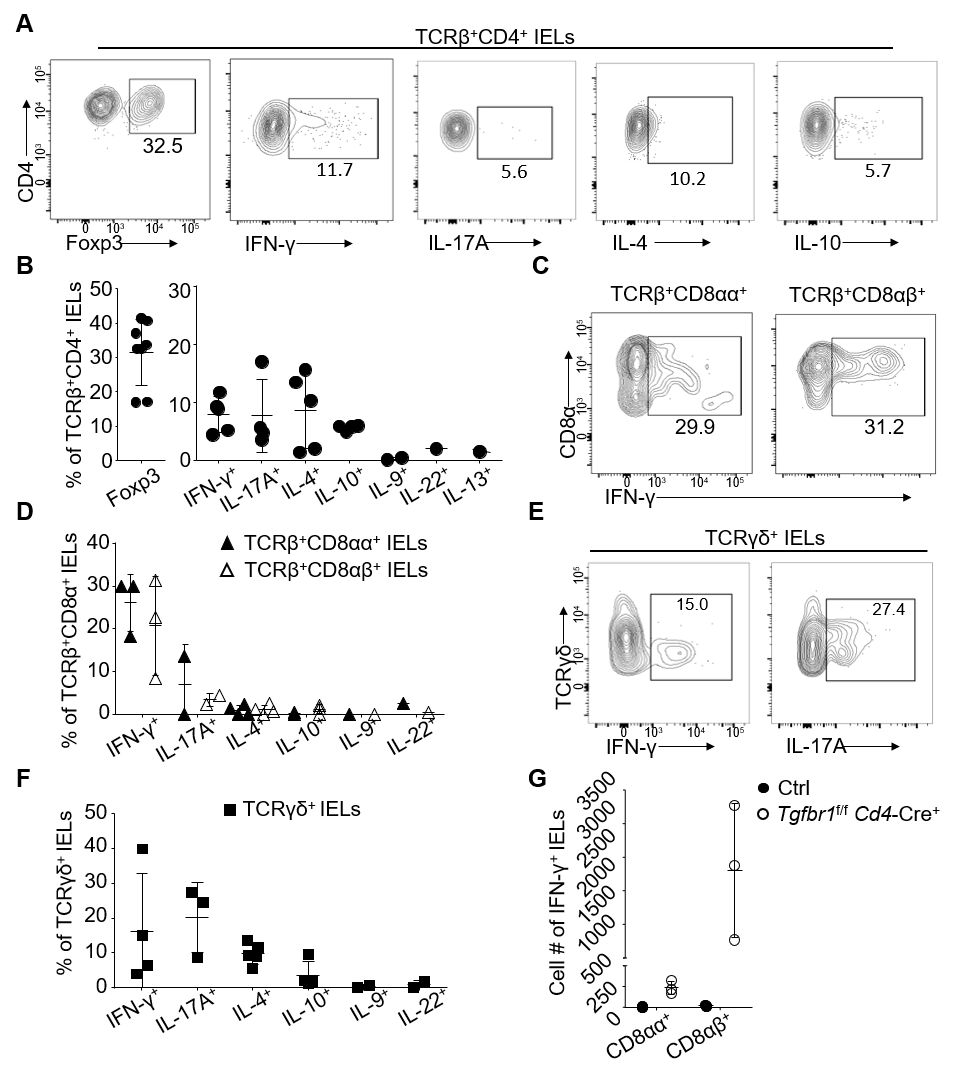

Supplement: Supplementary Figure 3 — IELs were isolated from 6- to 8-week-old C57BL/6 mice. Each dot in dot plots is representing individual experiment in which cells were isolated from two mice and pooled. Flow cytometric plots are representative of 4–7 independent experiments. (A) Representative plots of Foxp3, IFN-γ, IL-17A, IL-4, and IL-10 in TCRβ+CD4+ oral IELs. (B) Summarized data showing frequency of cytokine production from oral IELs. (C) Representative plots of TCRβ+CD8αα+IFN-γ+ or TCRβ+CD8αα+IFN-γ+ oral IELs. (D) Summarized data showing frequency of cytokine production from TCRβ+CD8αα+ or TCRβ+CD8αβ+ oral IELs. (E) Representative plots of TCRγδ+IFN-γ+ or TCRγδ+IL-17A+ oral IELs. (F) Summarized data showing frequency of cytokine production from TCRγδ+ oral IELs. (G) Cell number of IFN-γ+ IELs of TCRβ+CD8αα+ or TCRβ+CD8αβ+ subsets from Tgfbr1f/f Cd4-Cre+ mice and age matched littermate controls. [file Image_3.TIF]

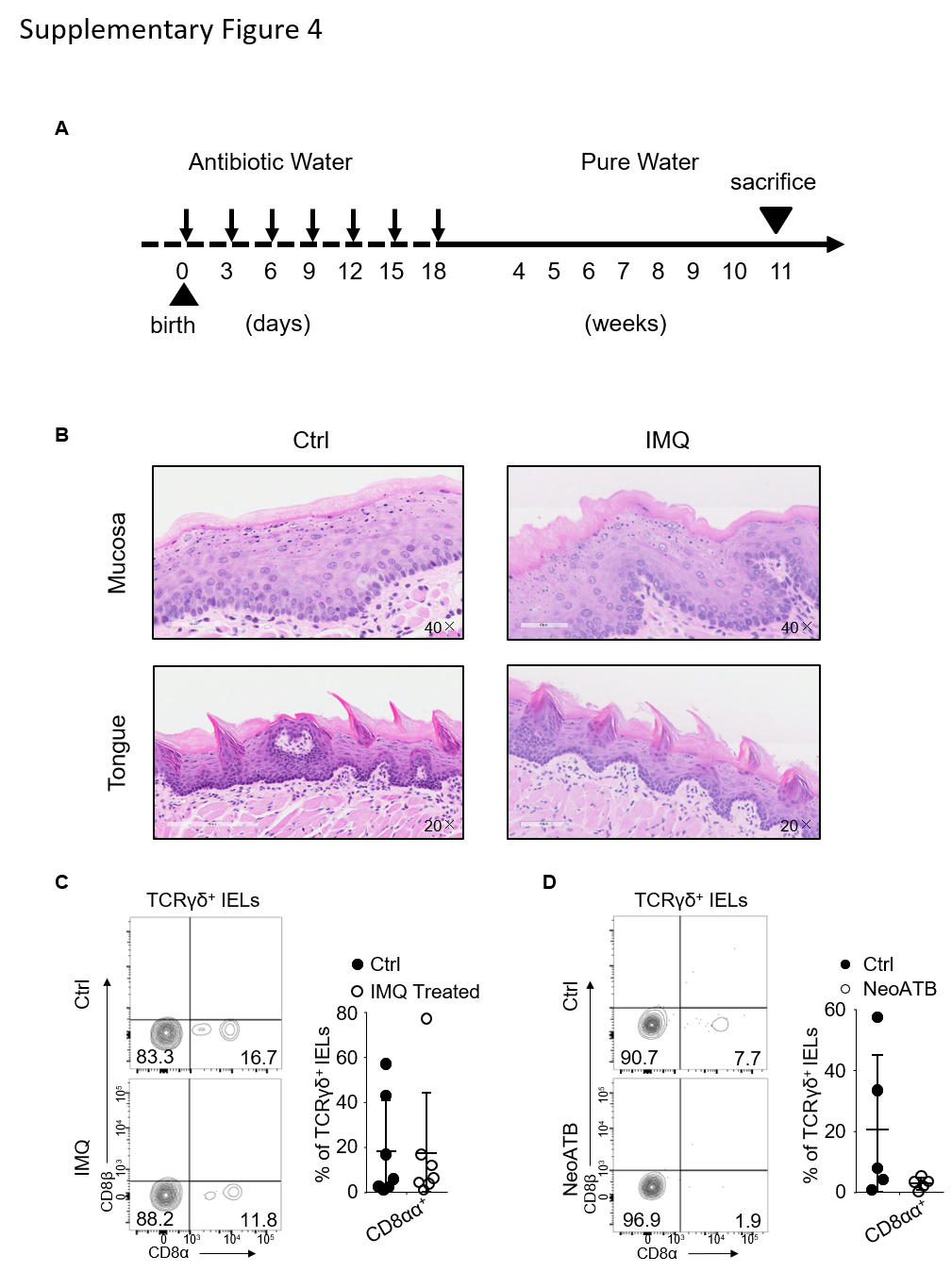

Supplement: Supplementary Figure 4 — (A) Immunization scheme of neonatal antibiotic treatment. (B) H&E stained buccal and tongue mucosa sections from Control or IMQ treated mice. (C) Representative flow cytometric plots of TCRγδ+CD8αα+ oral IELs from Control or IMQ treated mice. (D) Representative plots of TCRγδ+CD8αα+ oral IELs from Control or NeoATB mice. [file Image_4.TIF]
